# AdaptiveInvolutionNet: Spatially-adaptive involution with channel-wise attention for breast MRI tumor classification

**DOI:** 10.1371/journal.pone.0340808

**Published:** 2026-02-19

**Authors:** Saeed Alqahtani, Khaled Alqahtani, Faisal Alshomrani, Khaled AlQahtani

**Affiliations:** 1 Radiological Sciences Department, College of Applied Medical Sciences, Najran University, Najran, Kingdom of Saudi Arabia; 2 Biomedial Physics Department, King Fasial Hospital and Research Centre, Riyadh, Kingdom of Saudi Arabia; 3 Department of Diagnostic Radiology Technology, College of Applied Medical Science, Taibah University, Medinah, Kingdom of Saudi Arabia; 4 Radiology Department, King Abdulaziz Specialist Hospital, Taif, Kingdom of Saudi Arabia; Kafkas University: Kafkas Universitesi, TÜRKIYE

## Abstract

**Background:** Early and accurate classification of breast tumors from MRI scans is essential for improving patient outcomes. However, a key limitation of conventional deep learning models, such as Convolutional Neural Networks (CNNs), is their difficulty in capturing the subtle, spatially variant features that are crucial for precise medical image interpretation. **Objective:** To address this limitation, we propose a novel deep learning framework called AdaptiveInvolutionNet (AIN). This hybrid architecture is specifically designed to improve discriminative feature learning for breast tumor classification by integrating two key mechanisms: spatially-adaptive involution layers and channel-wise attention. **Methods:** Our AIN model employs a unique strategy for feature extraction. In its early layers, it utilizes spatially-adaptive involution kernels, which are highly effective at capturing fine-grained, localized features. As the network deepens, it transitions to conventional convolutions to maintain computational efficiency. To further enhance its diagnostic capabilities, we have embedded channel-wise attention mechanisms (specifically, squeeze-and-excitation modules) within the residual connections of the network. This allows the model to dynamically and selectively amplify diagnostically relevant features while suppressing less important ones. The model was rigorously trained and evaluated on a large, balanced dataset of 6,000 breast MRI images (3,000 benign, 3,000 malignant) using a robust five-fold cross-validation protocol. **Results:** AIN demonstrated superior performance, achieving a high test accuracy of 97%. This performance was consistent and reliable across all folds, with an average accuracy of 96% (± 1%). The model also showed strong agreement with true labels, indicated by a high Cohen’s Kappa score of 0.93 (± 0.01), and produced well-calibrated, trustworthy predictions with a low Brier score of just 0.0241. **Conclusion:** By successfully uniting an adaptive spatial feature extraction method with powerful attention mechanisms, AIN represents a significant advancement in medical image analysis. Its high accuracy, robust generalization, and consistent reliability demonstrate a strong potential for it to serve as a valuable and dependable computer-aided diagnostic tool for breast cancer detection in clinical settings.

## 1 Introduction

Breast cancer represents a major global health challenge. It has become the most commonly occurring cancer among women, affecting around 23% of all the women worldwide. More than 80% of the BC cases have been recorded in women with age of 45 years and more [1–3]. The causative factors of BC are multifactorial and very diverse in nature. These include hormonal imbalances, radiation exposure, genetic mutations (e.g., mutation in BRCA gene), obesity, and could be diet and life stress in general [4].

Mammography technique is currently the gold standard for the early detection of BC [5]. Mammography till date has proven to be the most capable method for identifying breast tumors before the development of the physical symptoms and thus significantly reducing mortality rates [6,7]. Mammography still has many limitations such as a low diagnostic yield and false negatives [8]. To overcome these challenges, a biopsy is always recommended for the lesions that have a greater chance (more than 2%) of being suspected with malignant tumors. Roughly less than 30% of these lesions have a proven malignancy [9]. To tackle the unnecessary problems with biopsy, another advanced approach, MRI (the Magnetic Resonance Imaging) has been adopted for BC detection [10,11].

MRI is an excellent detection approach because it has non-ionizing radiations and superior soft tissue resolution [12]. But, there are some limitations associated with MRI as well. For interpreting the MRI images, one needs highly skilled experts [13,14]. There are many cancer detecting methods formulated and evaluated recently. These methods utilized different types of risks aspects associated with imaging, genetics, and public health data. But these aspects are not sufficient for an individual to deal with one or more critical diagnostic screenings and correctly calculate the contingency of BC [15].

There are still some methods that rely solely on the variables such as breast density. Hence, these tools are also not appropriate for both the practitioners and the patients. Hence, there’s a growing need for automated, standardized, personalized screening approaches that have the ability to integrate genetic, imaging, and clinical data to better predict risk and guide early interventions. Recent advancements in the AI (Artificial Intelligence) technology have offered a significant improvement to the diagnostic performance of MRI in BC diagnosis and detection [16,17].

SVM (Support Vector Machine) is one of the most widely used ML (Machine Learning) based algorithm that uses supervised learning for BC diagnosis. The working principle of this technique includes working in high-dimensional space by finding the optimal hyper-plane for the separation of malignant and benign tumors. This approach is highly efficient where the dataset is small [18,19].

RF (Random Forest) model is another ensemble-based learning approach that is developed by combining multiple DT’s (Decision Trees) to get a stable, efficient and more accurate prediction [20,21]. RF method is known for its ability to reduce the over-fitting and handling missing data. It is highly effective for gene expression profiling and biopsy classification. K-Nearest Neighbors or denoted as KNN works by classifying the data points on the basis of categories of their nearest neighbors. It is a powerful approach for BC diagnosis. KNN can be optimized by combining with other feature selection methods. Based on the similarity measures, this method is highly efficient in identifying the tumor patterns [22]. Artificial Neural Networks (ANN) and NB (Naïve Bayes) are also some of the most commonly used ML approaches for BC detection [23–27].

ANNs have the ability to learn complex input and output relationships. In BC detection, this model takes the features such as texture, shape, size of the tumor from mammography, MRI, and histopathological data and defines nonlinear relationships between these features. NB is a probabilistic classifier that is based on the Bayes’ theorem. It detects the disease by assuming independence among the input features. It is highly efficient for binary classification problems mainly in the structured data [28]. Although these methods efficiently diagnosed BC, they still have some limitations. These approaches need a large, well-structured, hand-crafted dataset for training. As in these methods, the learning process is typically carried out via supervised learning; they require human supervision.

That’s where deep learning (DL) technology, a subfield of AI, having an ability to extract the complex patterns from large image data emerged [29]. Medical imaging, autonomous systems, and NLP (natural language processing) fields have been massively transformed due to the power DL methods to learn the hierarchical features automatically. This technology has not only enhanced the computational power but also works well with big data. In a recent study [30], the authors developed an automated DL model called CNN (Convolutional Neural Networks) for the efficient diagnosis of BC through the image texture attribute extraction.

Another research study [31] introduced MesoNet approach. This technique utilizes DL-based CNNs for the efficient prediction of the mesothelioma sufferers’ survival rates. MesoNet model didn’t require any toxicologist to manually locate the tag areas instead it used whole-slide digitalized images. Another powerful approach, SVM model well-trained on the CNN features was utilized on T2-Weighted (T2W) and DCEMRI images [32]. This study highlighted the fact that feature fusion approaches perform better than the image and classification fusion methods.

In another study [33], the authors proposed an advanced CNN architecture, called the boosted EfficientNet CNN. This model, as a solution to low image resolution, helped automatically detecting cancerous cells in the BC pathology tissues. Another DL-based classification system [34], the stacked sparse auto-encoders model was implemented for BC detection using the multi-parametric MRI. Many DL methods including CADNet157 [35], DCNN [36–38], deep fuzzy model [39], Gamma function based ensemble system [40], and hybrid rule-based systems [41] have been proposed for the efficient diagnosis of BC.

Some researchers found that the data augmentation technique can smooth down the training loss convergence[42–44]. Transfer learning and data augmentation can efficiently overcome the challenges of unavailability of medical imaging large datasets. Another study [45] led to the developed of DL-based system, known as the DenseNet121 CNN model. This method helped accurately identify the BC utilizing histopathology images. Another popular DL approach, the Vision Transformer (ViT) model was proposed [46]. This model, unlike the CNNs which solely focus on the local tissue lesions, utilizes self-attention learning to model global context and long-range dependencies. This method has proven to be an efficient approach for image classification tasks [47] , particularly in medical imaging, including breast cancer histopathological analysis [48–51], as it helps identify the spatial relationships and fine-grained features within the image.

He et al. [52] proposed a novel approach by integrating discrete wavelet transform (DWT) with a ViT, enabling the network to capture significant frequency-domain features from ultrasound images and thereby improving its receptive fields. Similarly, Jahan et al. [53] developed a comprehensive deep learning framework that utilizes a ViT-based model for both the detection of cancerous patches and the identification of cancer subtypes from whole slide images (WSIs), demonstrating superior performance compared to other deep learning models. In another study, Hayat et al. [54] introduced a hybrid model that combines the feature extraction capabilities of EfficientNetV2 with the classification power of a ViT, achieving high accuracy in classifying histopathological images. Addressing the limitations of standard ViTs in medical imaging, Babita and Nayak [55] designed the RDTNet, which incorporates a residual deformable attention-based transformer layer (RDTL) to capture both local and global contextual details from histopathological images.

DL has shown great potential for automated breast cancer detection from MRI. However, existing methods struggle to achieve the reliability needed for real clinical use. Current DL models use standard convolutions that process images in a fixed, uniform way. This approach has a major limitation—breast tumors come in many different shapes and sizes. Some are small and round, while others are large and irregular with unclear edges. Because these models cannot adapt to different tumor characteristics, they miss important details such as: Faint or blurry tumor boundaries, Small irregular patterns, and Subtle differences in tissue texture. This paper addresses this gap by proposing AdaptiveInvolutionNet (AIN), a new deep learning model designed specifically to adapt to different tumor characteristics in breast MRI.

- Novel Hybrid Architecture: Introduces AIN, the first breast MRI classification framework to combine spatially-adaptive involution layers with channel-wise attention, enabling precise spatial feature extraction and enhanced channel-wise feature recalibration.
- Improved Feature Learning: Incorporates learnable fusion of involution and convolutional pathways and progressive channel expansion, achieving superior representation of tumor boundaries and tissue heterogeneity.
- Robust Evaluation Protocol: Implements five-fold cross-validation with calibration curve analysis, ensuring well-calibrated probability estimates and reliable generalization across folds.
- High Performance: Demonstrates 97% accuracy and consistent reliability, highlighting AIN’s potential as a computer-aided diagnosis tool to support radiologists in early breast cancer detection.

These contributions advance medical image analysis by improving diagnostic accuracy for breast cancer detection.

The rest of the paper is organized as follows: Sect 2 describes the detailed material and methods used for this work. Sect 3 presents the model performance, which includes five-fold validation, an ablation study, and a comparative analysis. Finally, Sect 4 concludes the paper.

## 2 Materials and methods

### 2.1 Dataset detail

The dataset utilized in this study comprises breast Magnetic Resonance Imaging (MRI) scans, meticulously curated from The Cancer Imaging Archive (TCIA) Breast Diagnosis collection (https://www.cancerimagingarchive.net/collection/breast-diagnosis/). The original TCIA collection is extensive, encompassing over 101,000 medical images across diverse modalities, including MRI, Computed Tomography, Positron Emission Tomography, and Mammography. This comprehensive repository also includes associated clinical metadata, such as Diagnosis, Molecular Test results, Demographic information, Pathology Details, Radiomic Features, and Measurement data.

**Fig 1:**
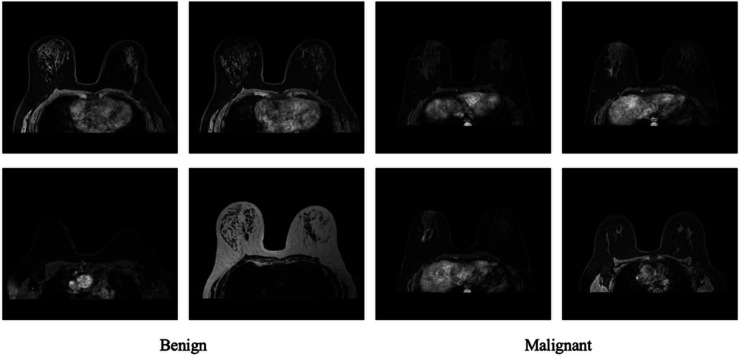
Collection of breast MRI scans, categorized into Benign and Malignant cases.

For the specific objectives of this research, only MRI images were retained, allowing for a focused investigation into breast tumor classification using this particular imaging modality. The core characteristics of the final dataset subset used for model training and evaluation are summarized in Table 1. Dataset sample images are shown in Fig 1.

**Table 1:** Summary of the TCIA BREAST-DIAGNOSIS dataset characteristics.

Characteristic	Description
Number of Subjects	88
Imaging Modalities	Magnetic Resonance (MR), Computed Tomography (CT),
	Positron Emission Tomography (PT), Mammography (MG)
Cancer Types	Breast Cancer (including high-risk normals, DCIS,
	fibroids, and lobular carcinomas)
Data Types	DICOM Images, Clinical Reports, Pathology Reports,
	Genomic Data (ER, PR, HER2, Oncotype DX)
Total Images	105,050
Total Size	60.87 GB
Total Samples Used	6,000 (3,000 Benign, 3,000 Malignant) MR Images
Output Task	Binary Classification (Benign vs. Malignant)
Data Source	The Cancer Imaging Archive (TCIA) [?]

### 2.2 Dataset preprocessing

The breast MRI images underwent a comprehensive series of preprocessing and augmentation steps to optimize them for deep learning model training and evaluation. These procedures were systematically managed through a custom class, designed to integrate seamlessly with PyTorch’s Data Loader for efficient batch processing.

Initially, images were loaded from a structured directory, where benign and malignant cases were organized into distinct folders. Upon loading, each image file was consistently converted to the RGB format. A critical initial check was implemented for image dimensions: if an image’s width or height was found to be less than 32 pixels, it was automatically resized to a standard 224×224 pixels. A stratified split, maintaining an 80/20 ratio (80% for training and 20% for testing), was performed.

For the **training data and validation (test) data**, a sequence of augmentations was applied to introduce variability and improve model robustness. Images were first resized to 256×256 pixels, followed by a random 224×224 pixel crop, which encouraged the model to learn features from various parts of the image. Further augmentation included random horizontal flipping with a 0.5 probability, random rotations up to 10 degrees, and minor color jittering (brightness, contrast, saturation at 0.1, hue at 0.05) to simulate diverse lighting conditions and scanner variations. Finally, these augmented images were converted into PyTorch tensors and normalized using standard mean and standard deviation values derived from the ImageNet dataset (mean=[0.485,0.456,0.406], std=[0.229,0.224,0.225]), a common practice for stabilizing training, especially with pre-trained models.

Finally, the preprocessed train_dataset and test_dataset instances were integrated with PyTorch’s Data Loader to facilitate efficient batch processing during model training and evaluation. A batch_size of 16 was configured for both loaders. The training loader was set to shuffle=True to randomize sample order in each epoch and drop_last=True to discard any incomplete final batches. Both data loaders leveraged num_workers=2 for parallel data loading and pin_memory=True to expedite data transfer to the GPU, optimizing overall training efficiency.

### 2.3 Comparison with other breast cancer datasets

We briefly compare the Breast MRI Tumor Classification Dataset (used in this study) with two prominent public benchmarks: the TCIA breast cancer MRI collections and the BUSI ultrasound dataset. These comparisons underscore the unique challenges and opportunities in multimodal breast imaging for AI-driven diagnostics.

The TCIA (The Cancer Imaging Archive) hosts multiple breast cancer collections, such as TCGA-BRCA (139 subjects with MRI linked to genomic data) and Duke-Breast-Cancer-MRI (922 pre-operative DCE-MRI cases), emphasizing large-scale, multi-institutional MRI data for radiogenomics and tumor phenotyping. Our dataset aligns closely with TCIA’s MRI focus, comprising 6,000 de-identified MRI slices (balanced benign/malignant) resized to 224×224, but it is more accessible via Kaggle for rapid prototyping without DICOM handling. In contrast, the BUSI (Breast Ultrasound Images) dataset contains 780 2D ultrasound scans (437 benign, 210 malignant, 133 normal) from 600 patients aged 25–75, collected in 2018 at Baheya Hospital (Egypt), with manual segmentation masks for tumor regions. BUSI supports classification and segmentation tasks in ultrasound, a cost-effective modality for dense breast screening, but lacks the volumetric depth and contrast enhancement of MRI.

### 2.4 Proposed model: AdaptiveInvolutionNet (AIN)

**Fig 2:**
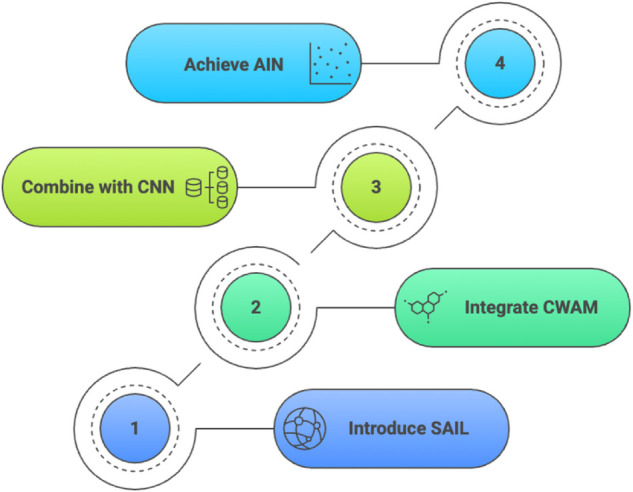
AIN Development Process - Sequential four-step development of Adaptive Involution Network for breast MRI tumor classification: introducing SAIL layers, integrating CWAM mechanisms, combining with CNN architecture, and achieving final AIN model.

The proposed model, AIN (Fig 2), introduces a novel deep learning architecture designed for breast MRI tumor classification. AIN synergistically integrates **Spatially-Adaptive Involution Layers** (SAIL) and **Channel-Wise Attention Mechanisms** (CWAM) within a residual learning framework to enhance feature representation and discrimination. By combining dynamic, content-aware involution operations with traditional convolutional feature extraction, AIN addresses the limitations of conventional convolutional neural networks (CNNs) in capturing spatially varying patterns and adaptive feature representations critical for medical image analysis. This section provides a detailed description of the architecture, its core components, and their mathematical formulations, ensuring clarity and rigor for research reproducibility.

The end-to-end transformation of the input image in AIN ${𝐗}_{\text{input}}\in {\mathbb{R}}^{B\times 3\times H\times W}$ to the final output ${𝐘}_{\text{final}}\in {\mathbb{R}}^{B\times N_{\text{classes}}}$ is mathematically expressed as Eq 1:

$${𝐘}_{\text{final}}=𝒞\left(𝒢\left({ℒ}_{4}\left({ℒ}_{3}\left({ℒ}_{2}\left({ℒ}_{1}\left(𝒮\left({𝐗}_{\text{input}}\right)\right)\right)\right)\right)\right)\right)$$ (1)

where:

- 𝒮(·): Stem block transformation for initial feature extraction.
- ${ℒ}_{i}(\cdot )$: Transformation at layer i∈{1,2,3,4}, incorporating SAIL or convolutional operations.
- 𝒢(·): Global feature aggregation via adaptive average pooling.
- 𝒞(·): Classification head transformation mapping features to class probabilities.

#### 2.4.1 Spatially-Adaptive Involution Layer (SAIL).

**Fig 3:**
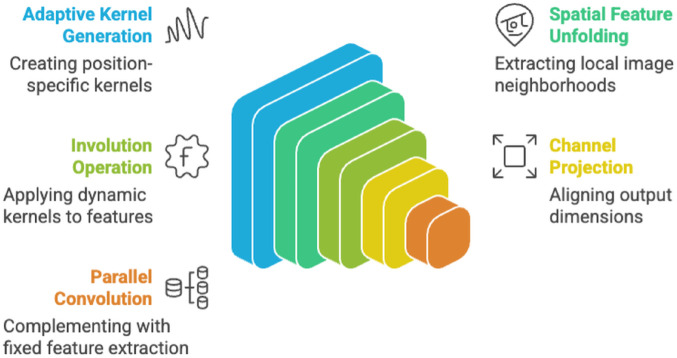
SAIL Architecture Overview - Diagram showing the key components of Spatially-Adaptive Involution Layers, including adaptive kernel generation, spatial feature unfolding, involution operations, channel projection, and parallel convolution across multi-scale feature maps.

The **Spatially-Adaptive Involution Layer (SAIL)** is the core innovation of AIN, designed to overcome the limitations of traditional CNN in capturing spatially varying patterns critical for breast MRI tumor classification as shown in Fig 3. Unlike conventional convolutions, which rely on fixed, translation-invariant kernels, SAIL dynamically generates location-specific kernels conditioned on the input feature content. This adaptivity enables the layer to respond to local image characteristics which are often heterogeneous in medical images. SAIL integrates a **learnable fusion coefficient** *α* to combine the outputs of an involution pathway and a parallel conventional convolution pathway, balancing flexibility with the stability of traditional feature extraction. By generating spatially adaptive kernels, SAIL enhances the model’s ability to capture fine-grained details while maintaining robustness across diverse image regions. This makes it particularly effective for tasks requiring precise localization and contextual understanding, such as distinguishing malignant from benign tumors in MRI scans.

The SAIL module consists of several sub-components: the **Adaptive Kernel Generation Network (AKGN)** synthesizes position-specific kernels through a bottleneck design, reducing computational overhead while ensuring rich kernel representations; **spatial feature unfolding** extracts local neighborhoods for pixel-wise operations; the **involution operation** applies these dynamic kernels to produce location-specific features; a **channel projection** aligns the output dimensions; and a **parallel convolution pathway** complements the involution process. The fusion mechanism, governed by *α*, is optimized end-to-end via gradient descent, allowing the model to learn the optimal balance between adaptive and fixed feature extraction. This design not only improves feature representation but also enhances the model’s adaptability to complex, spatially varying patterns in medical images.

For an input feature tensor $𝐗\in {\mathbb{R}}^{B\times C_{\text{in}}\times H\times W}$, where *B* is the batch size, $C_{\text{in}}$ is the number of input channels, and *H*,*W* are spatial dimensions, SAIL computes the output $𝐘\in {\mathbb{R}}^{B\times C_{\text{out}}\times H^{\prime }\times W^{\prime }}$ as Eq 2:

$$𝐘=\sigma (\alpha )⊙{𝐘}_{\text{inv}}+(1-\sigma (\alpha ))⊙{𝐘}_{\text{conv}}$$ (2)

where: *α* is initialized as a learnable scalar parameter with *α* = 0.0.

- $\sigma (\alpha )=\frac{1}{1+e^{-\alpha }}$: Sigmoid activation to constrain the fusion coefficient α∈ℝ to [0,1].
- ⊙: Element-wise Hadamard product.
- ${𝐘}_{\text{inv}}$: Output of the involution pathway.
- ${𝐘}_{\text{conv}}$: Output of the conventional convolution pathway.

The AKGN generates position-specific kernels as depicted in Eq 3:

$$𝐊=\sigma \left({𝐖}_{2}*\text{ReLU}\left({𝐖}_{1}*\text{GAP}(𝐗)\right)\right)$$ (3)

where $\text{GAP}(𝐗)=\frac{1}{H\times W}\sum_{i=1}^{H}\sum_{j=1}^{W}{𝐗}_{:,:,i,j}$, ${𝐖}_{1}\in {\mathbb{R}}^{C_{\text{in}}/r\times C_{\text{in}}\times 1\times 1}$, ${𝐖}_{2}\in {\mathbb{R}}^{K^{2}\times C_{\text{in}}/r\times 1\times 1}$, *r* is the channel reduction ratio (typically 4 or 8), and *K* is the kernel size (typically 3 or 5). The involution operation and other sub-components follow the original formulations.

#### 2.4.2 Channel-Wise Attention Mechanism (CWAM).

**Fig 4:**
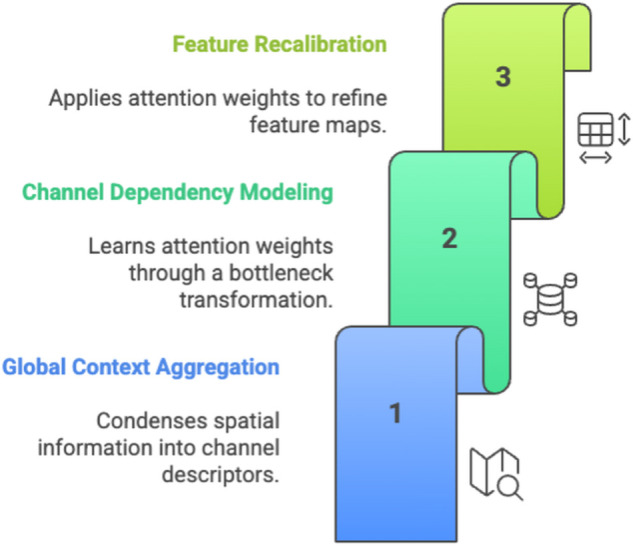
CWAM Three-Step Process - Channel-Wise Attention Mechanism showing global context aggregation, channel dependency modeling through bottleneck transformation, and feature recalibration with attention weights.

The **Channel-Wise Attention Mechanism (CWAM)** (Fig 4) is designed to enhance the discriminability of features by modeling inter-channel dependencies and recalibrating channel responses based on global context. In medical image analysis, CWAM ensures that the network prioritizes task-relevant features while suppressing irrelevant or noisy ones. By integrating a squeeze-and-excitation strategy, CWAM captures global spatial information through global average pooling (squeeze) and transforms it into channel-wise attention weights via a two-layer neural network (excitation). These weights are then applied to scale the input feature maps, effectively amplifying important channels and attenuating less relevant ones. This mechanism enhances the model’s ability to focus on diagnostically significant features, improving classification accuracy for breast MRI tumor differentiation.

The CWAM operates in three stages: **global context aggregation** condenses spatial information into channel descriptors; **channel dependency modeling** learns attention weights through a bottleneck transformation; and **feature recalibration** applies these weights to refine the feature maps. By embedding CWAM within the residual blocks, AIN ensures that attention-enhanced features are seamlessly integrated into the hierarchical feature learning process, contributing to robust and discriminative representations. The lightweight design of CWAM, achieved through channel reduction, maintains computational efficiency while significantly boosting feature quality.

For an input tensor $𝐔\in {\mathbb{R}}^{B\times C\times H\times W}$, the CWAM output is generated as shown in Eq 4:

$$\tilde{𝐔}=𝐔⊙𝐬$$ (4)

where $𝐬\in {\mathbb{R}}^{B\times C\times 1\times 1}$ are the attention weights, computed as Eq 5:

$$𝐬=\sigma \left({𝐖}_{2}\cdot \text{ReLU}\left({𝐖}_{1}\cdot \text{GAP}(𝐔)\right)\right)$$ (5)

where $\text{GAP}(𝐔)=\frac{1}{H\times W}\sum_{i=1}^{H}\sum_{j=1}^{W}{𝐔}_{:,c,i,j}$, ${𝐖}_{1}\in {\mathbb{R}}^{C/r\times C}$, ${𝐖}_{2}\in {\mathbb{R}}^{C\times C/r}$, and *r* is the reduction ratio (typically 16).

#### 2.4.3 Adaptive Residual Block (ARB).

**Fig 5:**
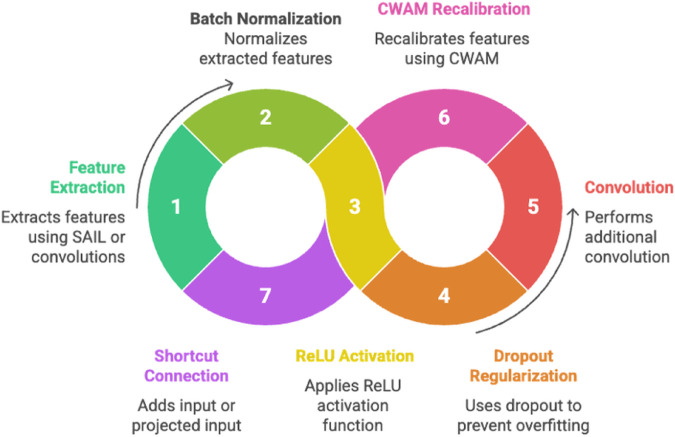
Adaptive Residual Block Flow - Circular process showing ARB components: feature extraction through SAIL/convolutions, batch normalization, ReLU activation, dropout regularization, additional convolution, CWAM recalibration, and shortcut connection.

The **Adaptive Residual Block (ARB)** (Fig 5) serves as the fundamental computational unit of AIN, integrating SAIL and CWAM within a residual learning framework to enable deep network training while mitigating the vanishing gradient problem. The ARB combines the adaptive feature extraction capabilities of SAIL (in early layers) or conventional convolutions (in deeper layers) with the feature recalibration of CWAM, ensuring that both local and global feature representations are optimized for the classification task. The residual structure, with its skip connections, facilitates gradient flow and allows the network to learn identity mappings when necessary, enhancing training stability and convergence. This is particularly crucial for deep architectures processing complex medical images, where maintaining gradient information across multiple layers is essential for capturing both low-level textures and high-level semantic patterns.

The ARB’s main transformation path includes a sequence of operations: either a SAIL or convolutional layer for feature extraction, followed by batch normalization, ReLU activation, dropout for regularization, a second convolution, and finally CWAM for channel-wise recalibration. The shortcut connection ensures that the input is either directly added (identity mapping) or projected to match the output dimensions, preserving information flow. By strategically deploying SAIL in early layers and transitioning to convolutions in deeper layers, the ARB balances computational efficiency with adaptive feature learning, making it well-suited for medical image analysis tasks requiring both precision and scalability.

The ARB output is defined as Eq 6:

𝐘=ReLU(ℱ(𝐗)+ℋ(𝐗)) (6)

where ℱ(𝐗) is the main transformation path is as Eq 7:

$$ℱ(𝐗)=\text{CWAM}\left({\text{BN}}_{2}\left({\text{Conv}}_{2}\left(\text{Dropout}\left(\text{ReLU}\left({\text{BN}}_{1}\left(𝒢(𝐗)\right)\right),p=0.1\right)\right)\right)\right)$$ (7)

and ℋ(𝐗) is the shortcut connection as depicted in Eq 8:

$$ℋ(𝐗)=\left\{ \begin{array}{cc} 𝐗 & \text{if }C_{\text{in}}=C_{\text{out}},s=1\\ {\text{BN}}_{\text{sh}}\left({𝐖}_{\text{sh}}*𝐗\right) & \text{otherwise} \end{array}\right.$$ (8)

where ${𝐖}_{\text{sh}}\in {\mathbb{R}}^{C_{\text{out}}\times C_{\text{in}}\times 1\times 1}$.

#### 2.4.4 AdaptiveInvolutionNet architecture.

**Fig 6:**
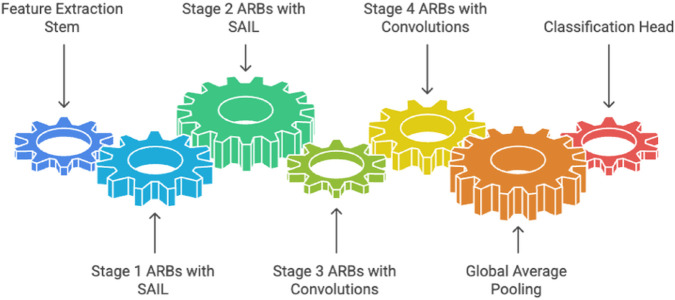
AIN Sequential Architecture - Gear-based diagram showing AIN processing stages from feature extraction stem through four ARB stages (SAIL-based stages 1-2, convolution-based stages 3-4), global average pooling, to classification head.

The **AdaptiveInvolutionNet (AIN)** architecture (Fig 6) is a hierarchical deep learning framework tailored for breast MRI tumor classification, leveraging the complementary strengths of SAIL, CWAM, and residual learning. AIN is structured to progressively extract features at increasing levels of abstraction, starting with fine-grained, spatially adaptive features in early layers and transitioning to high-level, computationally efficient feature representations in deeper layers. The architecture is composed of four main stages, each comprising multiple ARBs, with channel dimensions increasing (64, 128, 256, 512) and spatial resolutions decreasing through downsampling. This hierarchical design ensures that the network captures both local details (e.g., tumor textures) and global context (e.g., spatial relationships), critical for accurate medical image classification.

The AIN pipeline begins with a **feature extraction stem** that performs aggressive downsampling and initial feature extraction, followed by four stages of ARBs. In stages 1 and 2, ARBs incorporate SAIL to capture spatially adaptive features, enabling the model to focus on local variations in MRI images. In stages 3 and 4, ARBs use conventional convolutions to prioritize computational efficiency while maintaining robust feature generalization. The extracted features are aggregated via **global average pooling**, producing a compact representation that is fed into a **classification head**, a two-layer multi-layer perceptron (MLP) with dropout regularization to prevent overfitting. The strategic integration of SAIL in early layers and the transition to convolutions in deeper layers optimize the trade-off between adaptivity and scalability, making AIN a powerful architecture for complex medical imaging tasks.

#### 2.4.5 Architectural composition.

This section details the mathematical formulation and implementation specifics of AIN’s four primary architectural components, providing the technical foundation for system implementation and reproducibility.

**Feature extraction:** The Stem block performs initial feature extraction with aggressive spatial downsampling through a sequential combination of convolution, normalization, activation, and pooling operations. The stem block transformation is described as Eq 9:

$${𝐅}_{\text{stem}}={\text{MaxPool}}_{3\times 3}^{s=2,p=1}\left(\text{ReLU}\left(\text{BN}\left({𝐖}_{\text{stem}}{*}_{s=2,p=3}{𝐗}_{\text{in}}+{𝐛}_{\text{stem}}\right)\right)\right)$$ (9)

where:

- ${𝐖}_{\text{stem}}\in {\mathbb{R}}^{64\times 3\times 7\times 7}$: Convolution weights expanding input from 3 to 64 channels
- ${𝐛}_{\text{stem}}\in {\mathbb{R}}^{64}$: Channel-wise bias parameters
${*}_{s=2,p=3}$: Convolution with stride 2 and padding 3 for dimension preservation
- ${\text{MaxPool}}_{3\times 3}^{s=2,p=1}$: Max pooling with stride 2 and padding 1
- ${𝐅}_{\text{stem}}\in {\mathbb{R}}^{B\times 64\times H_{\text{stem}}\times W_{\text{stem}}}$: Output feature tensor
- $H_{\text{stem}}=\lfloor H_{\text{in}}/4\rfloor$, $W_{\text{stem}}=\lfloor W_{\text{in}}/4\rfloor$: Spatial dimensions after 4× reduction

**Hierarchical Feature Learning:** The network implements a four-stage hierarchical architecture with progressive feature abstraction. Each stage contains two ARBs with specific configurations as detailed in Eqs 10–13:

$${𝐅}_{1}={\text{ARB}}_{1,2}\left({\text{ARB}}_{1,1}\left({𝐅}_{\text{stem}}\right)\right)\in {\mathbb{R}}^{B\times 64\times H_{\text{stem}}\times W_{\text{stem}}}$$ (10)

$${𝐅}_{2}={\text{ARB}}_{2,2}\left({\text{ARB}}_{2,1}\left({𝐅}_{1}\right)\right)\in {\mathbb{R}}^{B\times 128\times H_{\text{stem}}/2\times W_{\text{stem}}/2}$$ (11)

$${𝐅}_{3}={\text{ARB}}_{3,2}\left({\text{ARB}}_{3,1}\left({𝐅}_{2}\right)\right)\in {\mathbb{R}}^{B\times 256\times H_{\text{stem}}/4\times W_{\text{stem}}/4}$$ (12)

$${𝐅}_{4}={\text{ARB}}_{4,2}\left({\text{ARB}}_{4,1}\left({𝐅}_{3}\right)\right)\in {\mathbb{R}}^{B\times 512\times H_{\text{stem}}/8\times W_{\text{stem}}/8}$$ (13)

Stage-specific configurations:

- **Stages 1-2**: Implement ARBs with SAIL modules for spatially-adaptive feature extraction
- **Stages 3-4**: Utilize conventional convolution-based ARBs for computational efficiency
- **Downsampling**: Applied at stage transitions (2, 3, 4) via stride-2 operations in the first ARB
- **Channel progression**: Doubles at each stage transition (64→128→256→512)

**Global Feature Aggregation:** It converts spatially distributed features into a fixed-size representation through adaptive average pooling as shown in Eq 14:

$${𝐅}_{\text{global}}={\text{AdaptiveAvgPool}}_{1\times 1}\left({𝐅}_{4}\right)=\frac{1}{H_{4}\times W_{4}}\sum_{i=1}^{H_{4}}\sum_{j=1}^{W_{4}}{𝐅}_{4}[:,:,i,j]$$ (14)

where $H_{4}=H_{\text{stem}}/8$, $W_{4}=W_{\text{stem}}/8$, and ${𝐅}_{\text{global}}\in {\mathbb{R}}^{B\times 512}$.

**Classification Head:** It implements a two-layer MLP with dropout regularization to map global features to class probabilities as shown in Eq 15:

$$𝐏=\text{softmax}\left({𝐖}_{\text{cls2}}\cdot \text{Dropout}\left(\text{ReLU}\left({𝐖}_{\text{cls1}}\cdot {𝐅}_{\text{global}}+{𝐛}_{\text{cls1}}\right),p=0.3\right)+{𝐛}_{\text{cls2}}\right)$$ (15)

where:

- ${𝐖}_{\text{cls1}}\in {\mathbb{R}}^{256\times 512}$, ${𝐛}_{\text{cls1}}\in {\mathbb{R}}^{256}$: First layer parameters
- ${𝐖}_{\text{cls2}}\in {\mathbb{R}}^{N_{\text{classes}}\times 256}$, ${𝐛}_{\text{cls2}}\in {\mathbb{R}}^{N_{\text{classes}}}$: Output layer parameters
- $𝐏\in {\mathbb{R}}^{B\times N_{\text{classes}}}$: Class probability distribution

The intermediate dimension of 256 provides sufficient representational capacity for task-specific transformations while maintaining computational efficiency.

#### 2.4.6 System model workflow.

The complete forward pass of AdaptiveInvolutionNet is summarized in Algorithm 1, which integrates the stem block, hierarchical ARB stages, global pooling, and classification head.

**Algorithm 1** AdaptiveInvolutionNet Forward Pass

**Require:** Input image ${𝐗}_{\text{in}}\in {\mathbb{R}}^{B\times 3\times H\times W}$

**Ensure:** Class probabilities $𝐏\in {\mathbb{R}}^{B\times N_{\text{classes}}}$

1: ${𝐅}_{0}\leftarrow \text{Stem}({𝐗}_{\text{in}})$

2:   **for** *i* = 1 to 4 **do**

3:    **for** *j* = 1 to 2 **do**

4:     **if** *i* ≤ 2 **then**

5:      ${𝐅}_{\text{i}}\leftarrow {\text{ARB-SAIL}}_{\text{i,j}}({𝐅}_{\text{i-1}})$

6:     **else**

7:      ${𝐅}_{\text{i}}\leftarrow {\text{ARB-Conv}}_{\text{i,j}}({𝐅}_{\text{i-1}})$

8:     **end if**

9:    **end for**

10:   **end for**

11: ${𝐅}_{\text{global}}\leftarrow \text{GlobalAvgPool}({𝐅}_{\text{4}})$

12: $𝐏\leftarrow \text{ClassificationHead}({𝐅}_{\text{global}})$ **return** 𝐏

The AdaptiveInvolutionNet architecture leverages the synergistic integration of SAIL, CWAM, and residual learning to achieve robust feature representation for breast MRI tumor classification. SAIL’s spatially adaptive kernels enable fine-grained feature extraction in early layers, capturing critical local patterns such as tumor boundaries. CWAM enhances feature discriminability by recalibrating channel responses, while the residual framework ensures stable training of deep networks. The strategic transition to conventional convolutions in deeper layers balances computational efficiency with generalization, making AIN suitable for complex medical image analysis tasks.

## 3 Result

### 3.1 Training environment

All training hyperparameters are detailed in Table 2 for full reproducibility. The model was trained and evaluated on a GPU-accelerated environment using torch.cuda, with automatic fallback to CPU if CUDA was unavailable.

**Table 2:** Complete list of training hyperparameters used for the proposed AIN model.

Hyperparameter	Value
Optimizer	Adam
Learning Rate (initial)	$1\times 10^{-4}$
Weight Decay	$1\times 10^{-4}$
Batch Size	16
Input Resolution	224×224
Data Augmentation	RandomCrop, HorizontalFlip, Rotation($\pm 10^{\circ }$), ColorJitter
Normalization	ImageNet mean/std
Loss Function	Cross-Entropy
Scheduler	StepLR (step=10, γ=0.5)
Max Epochs	100
Early Stopping Patience	15
Gradient Clipping	max_norm = 1.0
Dropout (classifier)	0.5, 0.3
Dropout2d (residual blocks)	0.1
Random Seed	42

### 3.2 Evaluation metrics definitions

**Precision** measures the proportion of true positive predictions among all positive predictions for a given class. It is defined as Eq 16:

$$\text{Precision}=\frac{\text{TP}}{\text{TP}+\text{FP}}$$ (16)

where TP is the number of true positives, and FP is the number of false positives.

**Recall** (or sensitivity) measures the proportion of true positive predictions among all actual positive instances. It is defined as as Eq 17:

$$\text{Recall}=\frac{\text{TP}}{\text{TP}+\text{FN}}$$ (17)

where FN is the number of false negatives.

**F1-Score** is the harmonic mean of precision and recall, providing a balanced measure of both metrics. It is defined as as Eq 18:

$$\text{F1-Score}=2\cdot \frac{\text{Precision}\cdot \text{Recall}}{\text{Precision}+\text{Recall}}$$ (18)

**Cohen’s Kappa** measures the agreement between predicted and actual classifications, accounting for chance agreement. It is defined as as Eq 19:

$$\kappa =\frac{p_{o}-p_{e}}{1-p_{e}}$$ (19)

where *p*_*o*_ is the observed agreement, and *p*_*e*_ is the expected agreement by chance.

**Matthews Correlation Coefficient (MCC)** is a balanced measure of classification performance, considering true and false positives and negatives. It is defined as as Eq 20:

$$\text{MCC}=\frac{\text{TP}\cdot \text{TN}-\text{FP}\cdot \text{FN}}{\sqrt{\left(\text{TP}+\text{FP})(\text{TP}+\text{FN})(\text{TN}+\text{FP})(\text{TN}+\text{FN}\right)}}$$ (20)

**ROC AUC Score** represents the area under the Receiver Operating Characteristic curve, measuring the model’s ability to distinguish between classes. A value of 1 indicates perfect discrimination, while 0.5 indicates no discrimination ability.

### 3.3 Training and validation loss-accuracy analysis

**Fig 7:**
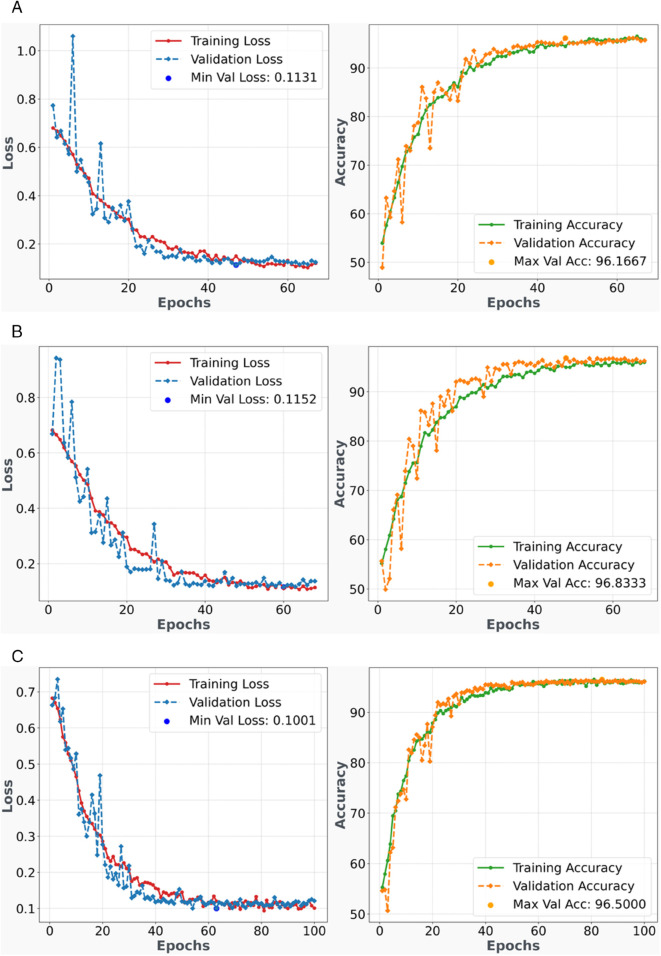
Training and Validation History - Folds 1-3 - Performance plots showing model accuracy and loss progression over training epochs for each fold during cross-validation.

**Fig 8:**
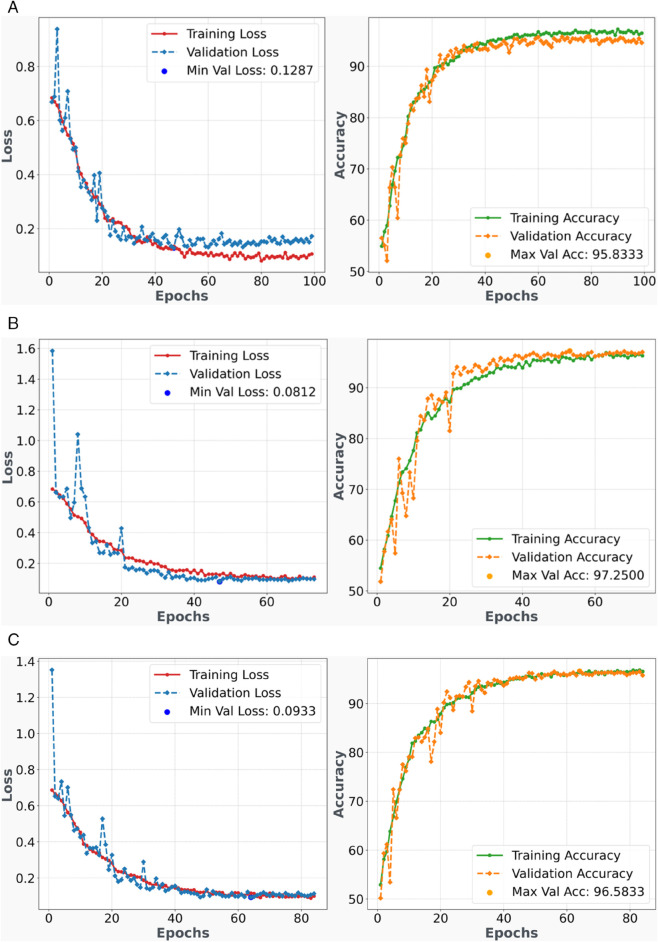
Training and Validation History - Folds 4-5 and Overall Performance - Performance plots showing model accuracy and loss progression over training epochs for the remaining cross-validation folds plus overall performance summary.

Figs 7 and 8 present comprehensive training history plots for the breast cancer classification model across five-fold cross-validation, illustrating the temporal evolution of model performance metrics including training/validation accuracy and loss functions over successive epochs. These visualizations provide critical insights into model convergence behavior, generalization capability, and potential overfitting or underfitting characteristics.

The training history for Fold 1 (Fig 7(a)) demonstrates robust convergence characteristics with synchronized improvement in both training and validation metrics. The accuracy curves exhibit steady upward progression, indicating effective gradient descent optimization and appropriate learning rate selection. The loss curves show corresponding downward trajectories with minimal oscillations, suggesting stable backpropagation dynamics and well-conditioned parameter updates. The close alignment between training and validation curves indicates balanced model complexity without significant overfitting, demonstrating good bias-variance trade-off optimization.

Fold 2 (Fig 7(b)) exhibits similar convergence patterns with consistent improvement across epochs. The training and validation accuracy curves maintain parallel progression, indicating robust generalization capability. The loss function demonstrates smooth monotonic decrease with minimal variance, suggesting effective optimization landscape navigation. The gap between training and validation metrics remains minimal, confirming the absence of significant overfitting and validating the regularization strategies employed.

The third fold (Fig 7(c)) shows excellent training stability with convergent behavior in both accuracy and loss metrics. The validation curves closely track the training curves, indicating strong model generalization across different data distributions. The smooth convergence pattern without significant plateauing suggests optimal learning rate scheduling and effective feature extraction capability throughout the training process.

Fold 4 (Fig 8(a)) demonstrates consistent training progression with stable convergence characteristics. The accuracy metrics show steady improvement with minimal validation-training gap, indicating robust generalization performance. The loss curves exhibit expected decreasing trends with controlled variance, suggesting effective gradient flow and parameter optimization. Any minor fluctuations in the validation curves reflect natural stochastic variations inherent in the training process rather than systematic overfitting issues.

The fifth fold (Fig 8(b)) exhibits optimal training characteristics with superior convergence behavior compared to other folds. The training and validation curves show excellent alignment with minimal divergence, indicating exceptional generalization capability. The loss function demonstrates rapid initial decrease followed by stable convergence, suggesting efficient optimization dynamics and well-tuned hyperparameters. This fold’s superior performance aligns with the confusion matrix analysis, confirming its status as the best-performing configuration.

The overall training history plot (Fig 8(c)) presents macro-averaged performance across all five folds, providing comprehensive insights into the model’s collective training dynamics. The aggregated loss curve demonstrates consistent downward progression with controlled variance, indicating robust optimization across different data partitions. The ensemble behavior validates the stability of the training protocol and confirms the effectiveness of the cross-validation framework.

### 3.4 Classification report analysis

Table 3 summarizes the classification performance of the proposed model for breast cancer classification using five-fold cross-validation. The table reports precision, recall, F1-score, and support for two classes (0: benign, 1: malignant) for each fold, alongside overall accuracy, macro average, and weighted average metrics. In Fold 1, the model achieves an accuracy of 0.96, with balanced precision and recall for both classes (0.95–0.97). Fold 2 maintains similar performance with an accuracy of 0.96 and slightly higher precision for class 1 (0.97). Fold 3 also reports an accuracy of 0.96, with class 1 achieving a high recall of 0.99. Fold 4 shows a slightly lower accuracy of 0.95, with marginally reduced F1-scores (0.95 for both classes). Fold 5 demonstrates the best performance, achieving the highest accuracy of 0.97, with consistent precision, recall, and F1-scores of 0.97 for both classes. The overall metrics across all folds yield an accuracy of 0.97, with macro and weighted averages of 0.97, reflecting robust performance. The support values indicate a balanced class distribution, with approximately 600 samples per class in the overall dataset. Based on these results, Fold 5 is identified as the best-performing fold due to its superior accuracy and balanced metrics, highlighting the model’s effectiveness in distinguishing benign and malignant cases.

**Table 3:** Classification report summary.

Fold	Class	Precision	Recall	F1-Score	Support
1	0	0.95	0.97	0.96	613
	1	0.97	0.94	0.96	587
	**Accuracy**	**0.96**			1200
	Macro Avg	0.96	0.96	0.96	1200
	Weighted Avg	0.96	0.96	0.96	1200
2	0	0.96	0.97	0.97	619
	1	0.97	0.95	0.96	581
	**Accuracy**	**0.96**			1200
	Macro Avg	0.96	0.96	0.96	1200
	Weighted Avg	0.96	0.96	0.96	1200
3	0	0.94	0.99	0.96	599
	1	0.99	0.93	0.96	601
	**Accuracy**	**0.96**			1200
	Macro Avg	0.96	0.96	0.96	1200
	Weighted Avg	0.96	0.96	0.96	1200
4	0	0.94	0.97	0.95	591
	1	0.97	0.94	0.95	609
	**Accuracy**	**0.95**			1200
	Macro Avg	0.95	0.95	0.95	1200
	Weighted Avg	0.95	0.95	0.95	1200
5	0	0.97	0.97	0.97	578
	1	0.97	0.97	0.97	622
	**Accuracy**	**0.97**			1200
	Macro Avg	0.97	0.97	0.97	1200
	Weighted Avg	0.97	0.97	0.97	1200
Overall	0	0.95	0.99	0.97	600
	1	0.99	0.94	0.97	600
	**Accuracy**	**0.97**			1200
	Macro Avg	0.97	0.97	0.97	1200
	Weighted Avg	0.97	0.97	0.97	1200

Table 4 presents the classification metrics for the proposed breast cancer classification model evaluated using five-fold cross-validation. The table reports Cohen’s Kappa, Matthews Correlation Coefficient (MCC), and ROC AUC Score for each fold, alongside their cross-validation averages and standard deviations. In Fold 1, the model achieves a Cohen’s Kappa of 0.9233, MCC of 0.9235, and ROC AUC of 0.9932, indicating strong agreement and discriminative power. Fold 2 shows improved performance with a Cohen’s Kappa of 0.9366, MCC of 0.9367, and ROC AUC of 0.9930. Fold 3 reports a Cohen’s Kappa of 0.9300, MCC of 0.9313, and a high ROC AUC of 0.9954. Fold 4 has slightly lower metrics, with a Cohen’s Kappa of 0.9167, MCC of 0.9173, and ROC AUC of 0.9927. Fold 5 demonstrates the best performance, achieving the highest Cohen’s Kappa and MCC of 0.9449 and a ROC AUC of 0.9964, reflecting superior model reliability and discrimination. The cross-validation summary indicates an average validation accuracy of 96.52% (± 0.50), with average Cohen’s Kappa of 0.9303 (± 0.0099), MCC of 0.9308 (± 0.0097), and ROC AUC of 0.9941 (± 0.0015). These results highlight the model’s robust and consistent performance across folds, with Fold 5 identified as the best-performing fold due to its highest metrics, underscoring the model’s effectiveness in classifying benign and malignant cases.

**Table 4:** Summary of classification metrics across five folds.

Fold	Cohen’s Kappa	Matthews Correlation Coefficient	ROC AUC Score
1/5	0.9233	0.9235	0.9932
2/5	0.9366	0.9367	0.9930
3/5	0.9300	0.9313	0.9954
4/5	0.9167	0.9173	0.9927
5/5	0.9449	0.9449	0.9964
**Cross-Validation Summary:**			
Average Validation Accuracy	96.52% ± 0.50		
Average Cohen’s Kappa	0.9303	± 0.0099	
Average MCC	0.9308	± 0.0097	
Average ROC AUC	0.9941	± 0.0015	

### 3.5 Confusion matrix analysis

**Fig 9:**
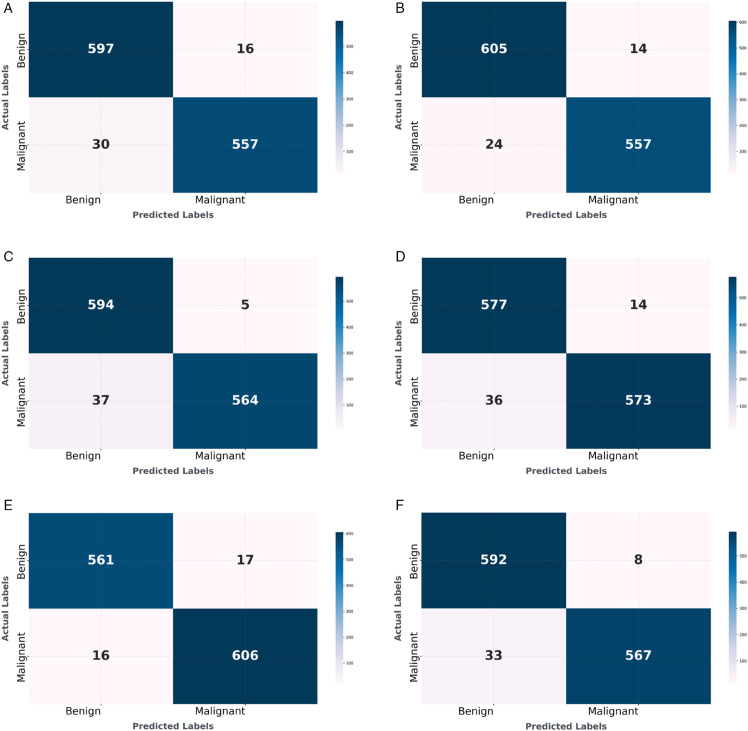
Detailed confusion matrices for breast cancer classification showing individual fold performance (a-e) and aggregated results across all folds (f), demonstrating model consistency and overall classification accuracy.

Fig 9 presents the confusion matrices for the breast cancer classification model evaluated using five-fold cross-validation. Subfigures (a) through (e) display the confusion matrices for Folds 1 to 5, respectively, detailing the true positives, true negatives, false positives, and false negatives for the two classes (0: benign, 1: malignant) across different data splits. Subfigure (f) shows the aggregated confusion matrix, summarizing the overall classification performance across all folds. Fold 1 demonstrates high true positive and true negative rates with minimal misclassifications. Fold 2 similarly shows strong classification performance with few errors. Fold 3 maintains robust performance, with balanced correct predictions for both classes. Fold 4 exhibits slightly more misclassifications compared to other folds. Fold 5 achieves the best performance, with the fewest misclassifications, indicating optimal classification of benign and malignant cases. The aggregated confusion matrix in Subfigure (f) confirms the model’s overall effectiveness, with balanced performance across both classes. Fold 5 is identified as the best-performing fold due to its minimal classification errors.

### 3.6 Calibration curve and brier score

**Fig 10:**
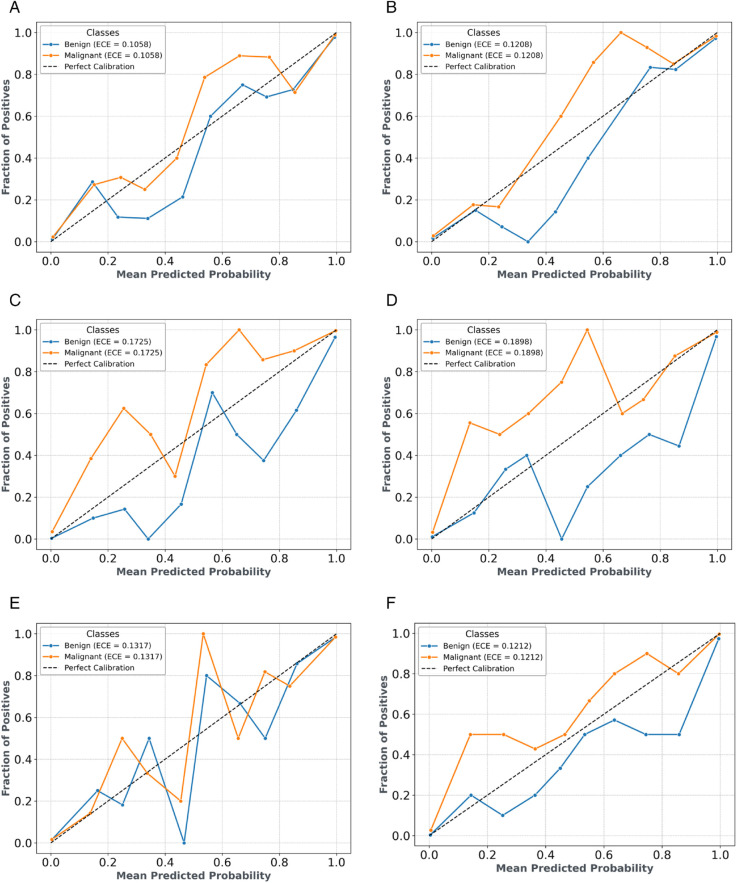
Calibration curves illustrating the reliability of predicted probabilities for different models within a five-fold cross-validation framework. Subfigures (a)–(e) show individual fold results, while (f) presents the overall calibration.

Fig 10 presents the calibration curves for the breast cancer classification model evaluated using five-fold cross-validation, illustrating the reliability of predicted probabilities for the two classes (0: benign, 1: malignant). Subfigures (a) through (e) display the calibration curves for Folds 1 to 5, respectively, showing the alignment between predicted probabilities and actual outcomes across different data splits. Subfigure (f) presents the aggregated calibration curve, summarizing the overall reliability across all folds. Fold 1 shows strong calibration with a Brier score of 0.0324, indicating good probability alignment. Fold 2 exhibits slightly better calibration with a Brier score of 0.0295, reflecting improved reliability. Fold 3 maintains robust calibration with a Brier score of 0.0300, showing consistent probability estimates. Fold 4 has a slightly higher Brier score of 0.0338, suggesting marginally less precise calibration. Fold 5 achieves the best calibration performance with the lowest Brier score of 0.0241, indicating the closest alignment between predicted probabilities and actual outcomes. The aggregated calibration curve in Subfigure (f) confirms the model’s overall robust probability estimates across both classes. Fold 5 is identified as the best-performing fold due to its lowest Brier score and superior calibration, highlighting the model’s effectiveness in producing reliable probability predictions for benign and malignant cases.

### 3.7 Ablation study

To validate the effectiveness of our architectural design choices, we conducted a comprehensive ablation study examining the contribution of each component. As shown in Table 5, the full model combining SAIL blocks in early layers, convolutional blocks in deeper layers, and Channel-Wise Attention Modules (CWAM) achieves the highest performance at 97.17% validation and 96.87% test accuracy. Removing SAIL entirely and using only convolutional layers results in a 0.70% performance drop (96.17%), while replacing all convolutional blocks with SAIL layers causes a more significant degradation to 94.08%, demonstrating that the hybrid architecture effectively leverages the complementary strengths of both approaches.

**Table 5:** Ablation study on the contribution of SAIL, CWAM (SE), and the hybrid layering strategy. All models are trained for a maximum of 50 epochs with early stopping (patience = 15).

Model	Val Acc (%)	Test Acc (%)
Full Model (SAIL+Conv+SE)	97.17	96.87
All-SAIL (pure)	94.08	94.08
All-Conv (no SAIL)	96.17	96.17
No CWAM (SE removed)	95.67	95.67
Only SAIL (early layers, no SE)	95.83	95.83
Only Conv (deep layers, SE kept)	94.50	94.50

The attention mechanism proves crucial for performance, as removing CWAM (SE) reduces accuracy to 95.67%. Furthermore, isolating components reveals that SAIL blocks alone in early layers without attention achieve 95.83%, while using only convolutional blocks in deep layers maintains 94.50%, confirming that both the strategic placement of SAIL in early feature extraction stages and the integration of attention mechanisms are essential. These results validate our hybrid layering strategy, where SAIL captures complex spectral-spatial patterns in shallow layers while conventional convolutions provide computational efficiency in deeper layers, with CWAM enhancing discriminative feature learning throughout the network.

**Fig 11:**
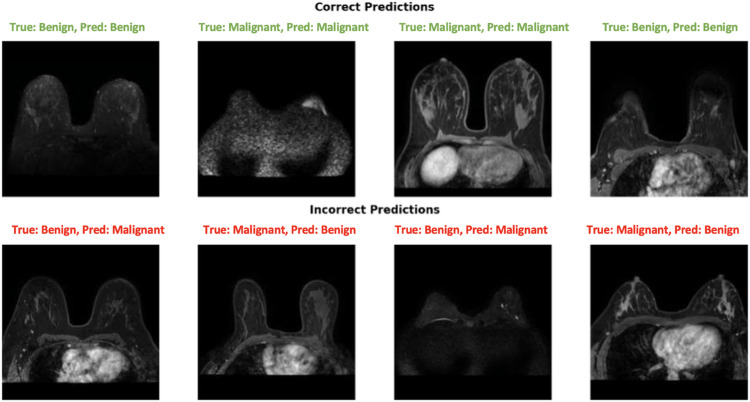
Qualitative analysis of model predictions.

Fig 11 illustrates both correct and incorrect predictions, including a clear example of a detected malignant tumor, thereby providing insight into the model’s decision-making and localization performance. The top row displays four examples of correct predictions (two benign, two malignant), with true labels, pred labels, and confidence scores in green. The bottom row shows four examples of incorrect predictions, with true labels, misclassified labels, and confidence scores in red.

### 3.8 Comparison with SOTA techniques

Table 6 presents a comprehensive performance comparison between the proposed AIN and existing state-of-the-art deep learning methods for breast cancer classification. The comparison demonstrates AIN’s superior performance across different architectural paradigms. The CNN-SVM hybrid approach by Edrees Almalki et al. achieved 93.6% accuracy by combining convolutional feature extraction with support vector machine classification, representing traditional machine learning integration with deep features. Chen et al.’s implementation of GoogLeNet, leveraging inception modules for multi-scale feature extraction, achieved 96.37% accuracy, demonstrating the effectiveness of multi-path architectures in medical imaging. Chaudhury et al.’s SqueezeNet implementation, designed for computational efficiency through fire modules, achieved 90.3% accuracy, highlighting the trade-off between model complexity and performance. The Inception-V3 CNN approach by Nadkarni et al. achieved 89.75% accuracy, utilizing deep inception architectures with auxiliary classifiers for improved gradient flow. In contrast, the proposed AIN achieves 97% accuracy, representing a notable improvement of 0.63% over the best-performing baseline (GoogLeNet) and significant margins over other approaches (3.4% over CNN-SVM, 6.7% over SqueezeNet, and 7.25% over Inception-V3). This superior performance can be attributed to AIN’s novel integration of spatially-adaptive involution layers that generate content-aware kernels for capturing fine-grained spatial variations in breast MRI images, combined with channel-wise attention mechanisms that enhance feature discriminability. The hybrid architecture’s strategic deployment of adaptive operations in early layers for spatial feature extraction, transitioning to conventional convolutions in deeper layers for semantic pattern recognition, enables optimal balance between adaptability and computational efficiency.

**Table 6:** Performance comparison of AdaptiveInvolutionNet with state-of-the-art methods for breast cancer classification.

References	Proposed Model	Accuracy (%)
[56]	CNN-SVM	93.6
[57]	GoogLeNet	96.37
[58]	Squeeze Net	90.3
[59]	Inception-V3 CNN	89.75
Proposed	AIN	97

## 4 Conclusion

This study presents AdaptiveInvolutionNet (AIN), a spatially-adaptive hybrid deep learning framework for breast MRI tumor classification. By leveraging involution layers for fine-grained spatial feature extraction, channel-wise attention for selective feature amplification, and residual connections for stable deep learning, AIN consistently outperforms conventional CNNs, achieving 97% test accuracy with robust cross-validation performance. The well-calibrated predictions and low Brier scores underscore its reliability for clinical applications.

Beyond accuracy, AIN has the potential to reduce diagnostic delays, assist radiologists in interpreting large MRI datasets, and minimize false positives and negatives in breast cancer screening. However, the model’s evaluation was limited to a single dataset; future work will involve testing on multi-center, heterogeneous datasets, integrating multi-modal imaging (e.g., mammography, ultrasound), and exploring real-time deployment in clinical workflows. These future directions will further establish AIN as a practical and scalable tool for automated breast cancer diagnosis.
